# Assessing phenotype order in molecular data

**DOI:** 10.1038/s41598-019-48150-z

**Published:** 2019-08-13

**Authors:** Ludwig Lausser, Lisa M. Schäfer, Lyn-Rouven Schirra, Robin Szekely, Florian Schmid, Hans A. Kestler

**Affiliations:** Institute of Medical Systems Biology, Ulm University, 89069 Ulm, Germany

**Keywords:** Systems biology, Machine learning

## Abstract

Biological entities are key elements of biomedical research. Their definition and their relationships are important in areas such as phylogenetic reconstruction, developmental processes or tumor evolution. Hypotheses about relationships like phenotype order are often postulated based on prior knowledge or belief. Evidence on a molecular level is typically unknown and whether total orders are reflected in the molecular measurements is unclear or not assessed. In this work we propose a method that allows a fast and exhaustive screening for total orders in large datasets. We utilise ordinal classifier cascades to identify discriminable molecular representations of the phenotypes. These classifiers are constrained by an order hypothesis and are highly sensitive to incorrect assumptions. Two new error bounds, which are introduced and theoretically proven, lead to a substantial speed-up and allow the application to large collections of many phenotypes. In our experiments we show that by exhaustively evaluating all possible candidate orders, we are able to identify phenotype orders that best coincide with the high-dimensional molecular profiles.

## Introduction

Assessing the correspondence between observable phenotypes and their underlying molecular background is a challenging task in molecular biology. Even for pairwise comparisons it is not straight forward to confirm hypothesised relations in high-dimensional marker representations.

**Figure 1:**
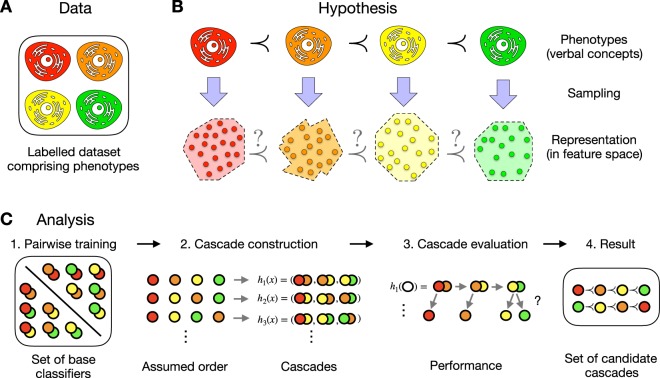
Assessment of phenotype order in molecular data (four phenotype/class example). (**A**) (Data): The data consists of a set of molecular measurements labelled with their phenotype names. (**B**) (Hypothesis): Is an ordinal relation on the phenotypes reflected in feature space? A trend in observable characteristics of phenotypes often induces hypotheses about ordinality on their molecular background. Its confirmation requires a reflection in the corresponding feature representation. (**C**) (Analysis): The analysis incorporates four different steps and evaluates which hypotheses are reflected in the specific feature representation. First, for all pairwise combinations binary base classifiers are trained in a pairwise manner. Second, for each possible order a cascade is built using the previously trained base classifiers. Third, the performance of these ensemble classifiers is analysed. The cascade classifier analyses each sample in a stepwise manner. For each base classifier it holds that if the sample belongs to its first class it is labelled and if not it is transferred to the next classifier. By doing so, regions of the feature space are defined and labelled dependent on the order of the base classifiers’ first class. Finally, a set of possible candidate cascades that show a good performance measured by the class-wise minimal sensitivity is returned.

This becomes even more evident for higher order relations among multiple phenotypes. In this case, local events and global processes might be confused as they both can lead to the same pattern of observable phenotypes. While local - pairwise - events might be reflected by any type of pairwise differences, an overall connecting pattern is required for global processes. An example, for such higher level relations are ordinal phenotypes of type $phenotyp{e}_{1}\,\prec \,phenotyp{e}_{2}\,\prec \,phenotyp{e}_{3}$ as they might occur in developmental processes^1–4^, like embryogenesis^5^, phylogenetic reconstruction^6–8^ or diagnostic stagings or gradings^9–12^. Their observable representations suggest an order of the phenotypes ($\prec$), which might lead to hypotheses on a connecting “ordinal” relation or process on a molecular level (Fig. 1B). Providing evidence for these hypotheses is quite challenging due to the high dimensionality of molecular profiles. Being defined for univariate categorical variables, the concept of ordinality can be embedded in many different ways in a multivariate real-valued feature representation. There might also be several ordinal relations that coexist in parallel.

In the research field of ordinal classification usually, a known order is used to improve classification performance. The assumption is that the given order between the classes (phenotypes) can be mapped to the given representation and hence also holds in the feature space. In this work, we instead propose a method that can check whether the reflection is provided, by elaborating a performance-based criterion for detecting and comparing ordinal structures in multivariate feature representations. We present an algorithm (CASCADES) that allows for systematic and exhaustive screens through the search space of all phenotype orders. It is applicable for extracting a small set of candidate orders from a feature representation that fulfils a minimal generalisation ability of a predictive model. Based on supervised classification, our method uses the canonical paradigm for learning relationships between raw uninterpreted feature representations and semantically meaningful phenotypes (classes, categories, concepts, etc.)^13–15^. Utilising feature representations and class memberships these techniques allow the extraction of phenotype-specific patterns and the construction of phenotype separating boundaries. In this way, classifiers identify characteristics of phenotypes or even learn the key attributes of their concepts. Mainly designed for discrimination the learning processes of classification algorithms often neglect the semantic relationships among classes. Standard training algorithms would neither request nor reconstruct such dependencies explicitly^16–18^.

For this reason, we focus on ordinal classifier cascades^19^ of binary base classifiers. They are a specialisation of general decision lists^20–22^. A predefined order of phenotypes constrains the learning algorithm of an ordinal classifier cascade. Initially designed for guiding the learning process, we showed that wrong assumptions on the class order can lead to severely decreased detection rates of an ordinal classifier cascade^23^.

In our approach (Fig. 1), we separate the training and evaluation of binary base classifiers from the construction of the classifier cascade. For the training, no order information is used, and each base classifier is trained independently. But the assumed phenotype order defines the evaluation sequence of these pairwise base classifiers. In each step of the evaluation a feature space region is labelled as decision region for a specific class, and the remaining space stays unlabelled. If the order is wrong, then samples of classes that are later in the order already lie within this region, whereas if it reflects the order they lie within the unlabelled region. Although trained only pairwise, the base classifiers show good performance when used to distinguish between a class and all following classes in the order.

Here, we utilise this susceptibility as a clear cut criterion for discriminating between class orders that allow a high generalisation ability or not. We provide theoretical upper bounds on the class-wise sensitivities of ordinal classifier cascades, which would enable the proposed algorithm to scale up to large collections of phenotypes. The combination of the pairwise training scheme and these bounds lead to a complexity reduction, as the number of base classifier trainings, in a single train-test experiment, for *n* classes is reduced from (*n* − 1)*n*! to (*n* − 1)*n*, and the number of cascade constructions and trainings is in the worst case *n*! but decreases by the number of cascades that do not pass this bound. We could show the utility of our method to identify reflected orderings in experimental evaluations on artificial data and gene expression profiles of developmental and ageing phenotypes.

## Results

We evaluated the ability of the CASCADES algorithm to detect reflected orders in feature space based on artificial and existing gene expression datasets (see Methods). For our analysis a linear support vector machine (SVM)^18^ was chosen as a base classifier for the ordinal cascades due to its superior performance^23^. The SVM was imported from the LIBSVM library^24^. Its cost parameter was fixed to a value of one.

The performance of the ordinal cascades as well as their base classifiers were evaluated in 10 × 10 cross-validation (CV) experiments^25^. The 10 × 10 CV experiments were repeated for all class orders ($|{\mathscr{Y}}|!$ experiments) and the performance was measured in terms of minimal class-wise sensitivity. All classification experiments were performed with help of the TunePareto software^26^.

### Artificial datasets

We performed experiments on three different kinds of artificial datasets, two of them reflect an ordered sequence of sample clouds in the feature space (*d*_1_ and *d*_2_) and one does not (*d*_3_) (Supplementary Fig. S1). Each dataset comprises $|{\mathscr{Y}}|=10$ classes (*i* = 1, …, 10) of 100 samples. The instances of class *y* are drawn independent and identically distributed from a normal distribution ${\bf{x}} \sim {\mathscr{N}}({{\bf{x}}}_{y},sd)$ in dependence of a class specific centroid **m**_*y*_ ∈ ℝ^2^. The standard deviation *sd* was identical for all classes. Experiments were performed for standard deviations *sd* ∈ {0.1, 0.2, …, 1.0}.

The analysis on the simple artificial datasets show that our method can distinguish between phenotype orders that are reflected and are not reflected in the two dimensional data. The ordinal assumption is imposed by a common increase in both features. The results for *sd* = 0.2 are given in Table 1. It can be seen that for *d*_1_ and *d*_2_ the correct order and its inverse are returned. All other possible orders show a minimal class-wise sensitivity lower than 50%. For *d*_3_ (non-ordinal) no order passed the threshold of 50%.

**Table 1:** Evaluation of the CASCADES algorithm on the artificial datasets *d*_1_, …, *d*_3_ and the real datasets *d*_4_, …, *d*_9_.

Id	Name	Ordinal Cascades	Bound	Min Sens.
**d**_**1**_ **:**	**linear** (10!−2 cascades rejected)	(a) ${y}_{1}\,\prec \,\ldots \,\prec \,{y}_{10}$	**100%**	**100%**
		(b) ${y}_{10}\,\prec \,\ldots \,\prec \,{y}_{11}$	**100%**	**100%**
**d**_**2**_ **:**	**curved** (10!−2 cascades rejected)	(a) ${y}_{1}\,\prec \,\ldots \,\prec \,{y}_{10}$	**100%**	**100%**
		(b) ${y}_{10}\,\prec \,\ldots \,\prec \,{y}_{1}$	**100%**	**100%**
**d**_**3**_ **:**	**non-ordinal** (all cascades rejected)	—	—	—
**d**_**4**_ **:**	**Drosophila melanogaster** (21 cascades rejected)	(a) $embryo\,\prec \,larva\,\prec \,pupa\,\prec \,adult$	**94.4%**	**89.4%**
		(b) $adult\,\prec \,pupa\,\prec \,larva\,\prec \,embryo$	72.3%	72.3%
		(c) $adult\,\prec \,pupa\,\prec \,embryo\,\prec \,larva$	71.0%	71.0%
**d**_**5**_ **:**	**Danio rerio** (117 cascades rejected)	(a) $adul{t}_{2}\,\prec \,adul{t}_{1}\,\prec \,embry{o}_{3}\,\prec \,embry{o}_{2}\,\prec \,embry{o}_{1}$	**85.0%**	**85.0%**
		(b) $adul{t}_{2}\,\prec \,adul{t}_{1}\,\prec \,embry{o}_{3}\,\prec \,embry{o}_{1}\,\prec \,embry{o}_{2}$	**85.0%**	**85.0%**
		(c) $adul{t}_{2}\,\prec \,adul{t}_{1}\,\prec \,embry{o}_{1}\,\prec \,embry{o}_{2}\,\prec \,embry{o}_{3}$	54.7%	54.7%
**d**_**6**_ **:**	**Human muscle adaptation** (20 cascades rejected)	(a) $ag{e}_{1}\,\prec \,ag{e}_{2}\,\prec \,ag{e}_{3}\,\prec \,ag{e}_{4}$	**73.1%**	**62.5%**
		(b) $ag{e}_{1}\,\prec \,ag{e}_{2}\,\prec \,ag{e}_{4}\,\prec \,ag{e}_{3}$	**73.1%**	**62.5%**
		(c) $ag{e}_{3}\,\prec \,ag{e}_{4}\,\prec \,ag{e}_{2}\,\prec \,ag{e}_{1}$	50.5%	46.1%
		(d) $ag{e}_{3}\,\prec \,ag{e}_{4}\,\prec \,ag{e}_{1}\,\prec \,ag{e}_{2}$	50.5%	31.6%
**d**_**7**_ **:**	**Caenorhabditis elegans, stages** (116 cascades rejected)	(a) $stag{e}_{1}\,\prec \,stag{e}_{2}\,\prec \,stag{e}_{3}\,\prec \,stag{e}_{4}\,\prec \,stag{e}_{5}$	**91.7%**	**91.7%**
		(b) $stag{e}_{5}\,\prec \,stag{e}_{4}\,\prec \,stag{e}_{3}\,\prec \,stag{e}_{2}\,\prec \,stag{e}_{1}$	75.0%	66.7%
		(c) $stag{e}_{1}\,\prec \,stag{e}_{2}\,\prec \,stag{e}_{3}\,\prec \,stag{e}_{5}\,\prec \,stag{e}_{4}$	58.6%	58.6%
		(d) $stag{e}_{5}\,\prec \,stag{e}_{4}\,\prec \,stag{e}_{3}\,\prec \,stag{e}_{1}\,\prec \,stag{e}_{2}$	50.0%	50.0%
**d**_**8**_ **:**	**Caenorhabditis elegans, time** (10!−1 cascades rejected)	(a) ${t}_{1}\prec {t}_{2}\,\prec \,{t}_{3}\prec {t}_{4}\,\prec \,{t}_{5}\prec {t}_{6}\prec {t}_{7}\,\prec \,{t}_{8}\prec {t}_{9}\,\prec \,{t}_{10}$	**66.7%**	**66.7%**
**d**_**9**_ **:**	**Various cancer cell lines** (all cascades rejected)	—	—	—

Screening experiments were performed for sensitivity thresholds *t* ∈ {1.00,0.95, …, 0.50}. Each screening experiment comprises all $|{\mathscr{Y}}|!$ class orders. All candidate cascades that pass a minimal sensitivity threshold bound *t* ≥ 0.5 are reported. The bound and the real minimal class-wise sensitivities are reported. The highest bounds and minimal class-wise sensitivities are marked in boldface.

We additionally analysed the performance in dependency of the standard deviation of the artificial data clouds. For the dataset *d*_1_ (linear) and *d*_2_ (curved) the sensitivities under the correct assumption decline with increasing standard deviations (Supplementary Fig. S2). The corresponding bounds lie above the real sensitivities. For the wrong order, the sensitivity of at least one class drops. In the given example the sensitivities of classes *y*_1_ to *y*_5_ and the corresponding bounds are mainly identical to those of the correct class order but largest changes can be observed for classes *y*_6_ and *y*_7_. For the non-ordinal dataset *d*_3_ the minimal class-wise sensitivity is zero independent of the standard deviation (Supplementary Fig. S2).

For each dataset and each setting, all 10! ≈ 3.6⋅10^6^ possible class orders are screened and the number of remaining class orders is reported (Supplementary Fig. S3). Datasets *d*_1_ and *d*_2_ show comparable results. Our method returned at most four candidate cascades for each experiment. With increasing standard deviation the distinction of classes became harder. Candidate cascades could only fulfil lower sensitivity thresholds *t*. The bounds of all rejected cascades predicted minimal sensitivities below 0.5. With lower thresholds the chance of finding more than two candidate cascades increased. As expected, no candidates were proposed for dataset *d*_3_ (non-ordinal). Evaluating the real minimal class-wise sensitivities of the remaining cascades revealed that additional candidates were rejected.

### Gene expression datasets

Furthermore, experiments on existing gene expression data were performed (see Methods). We chose ordinal multi-class expression data for which the classes correspond to specific points in time of a process. In three datasets (*d*_4_, *d*_5_, *d*_7_) the classes correspond to developmental stages of *Drosophila melanogaster*^1^, *Danio rerio*^2^, and *Caenorhabditis elegans*^4^. Additionally, *d*_7_ was used with a different labelling. This further labelling was not based on stages but based on the point in time at which the sample was taken. The further dataset in our analysis *d*_6_ comprises transcriptome samples of human muscles^3^. The data was categorised into four classes according to the age (in years) of the participants. For all these datasets it is expected that the assumed order based on the order of points in time is reflected within the expression profiles. To test our method (CASCADES algorithm) on real data for which no order is assumed, we included gene expression profiles from cell lines that are derived from 9 different cancer tissue types^27^.

The results of the real datasets are shown in Table 1. As the performance of the cascade depends on a sensitivity bound which depends only on the performance of the independent base classifiers, first those candidate cascades are reported that pass a sensitivity bound *t* ≤ 0.5. For the temporal ordinal datasets *d*_4_–*d*_8_, the CASCADES algorithm rejected at least 83.3% of all candidate cascades. No candidate passed the CASCADES algorithm for the non-ordinal dataset *d*_9_. The number of candidates is further depleted by analysing the minimal class-wise sensitivity of the full cascades. For dataset *d*_4_, the highest minimal class-wise sensitivity (89.4%) was achieved by the correct class order. It was followed by the correct inverse class order (72.3%) and an incorrect class order (71.0%).

Three candidates passed the CASCADES algorithm for dataset *d*_5_. The highest minimal class-wise sensitivity was achieved by two candidate cascades (85.0%). Both reflect the inverse class order. The first one corresponds to the correct inverse class order. The second one assumes an incorrect class order $embry{o}_{3}\,\prec \,embry{o}_{1}\,\prec \,embry{o}_{2}$. The third candidate achieved a minimal class-wise sensitivity of 54.7%. A general division between the adult and embryo samples can be observed. The order might be explained by the duration between the different states. Whereas all three embryonic samples cover a range of 10 days after birth, the first adult class comprises samples taken at month 3 and the *adult*_2_ class is 1–2 years after birth. As a result the order assumption $embry{o}_{1}\,\prec \,embry{o}_{2}\,\prec \,embry{o}_{3}$ might only be reflected ambiguously.

Four class orders passed the CASCADES algorithm on dataset *d*_6_. Two of these candidates dropped out due to a minimal class-wise sensitivity lower than 50.0%. The remaining two achieved minimal class-wise sensitivities of 62.5%. One of these class orders corresponds to the correct class order. The second one proposes a partially consistent class order ($ag{e}_{4}\prec ag{e}_{3}$). As *age*_3_ and *age*_4_ comprise 10 years each and *age*_1_ and *age*_2_ 20 years it can be argued, similar to the cascades of *d*_5_. The two classes *age*_3_ and *age*_4_ might be too similar under the order assumption leading to comparable results.

For dataset *d*_7_, the minimal class-wise sensitivity of the correct class order outperformed the performance of other candidate cascades (91.7%). All other candidate cascades achieved a minimal class-wise sensitivity of at most 66.7%. Only one candidate cascade passed the CASCADES algorithm when analysed on the level of points in time (*d*_8_). The correct class order gained a minimal class-wise sensitivity of 66.7%, which was achieved for class *t*_2_. All other classes achieved class-wise sensitivities of at least 80.0%. Especially this dataset shows that our analysis does not aim at improving the classification performance as much as possible, but rather finding the order that outperforms other orders and this independent of a specific performance, as long as the performance is better than 50%.

## Discussion

Ordinal relations between phenotypes are often defined on a semantic level. These relations are assumed to be reflected in a given feature representation without evaluating whether these assumptions hold. It might be the case that independent causes lead to ordinal phenotype characteristics or that the order is not reflected in the chosen feature space because the measured features are not responsible for the observed order.

In this work, we present ordinal classification as an example of a supervised learning task that incorporates semantic relations in the training process of classification models. By constraining the learning process, ordinal classification results in a restricted model class, which is no longer able to separate an arbitrary landscape of classes. This property is used to falsify wrong assumptions on the dependencies of the classes and the chosen feature space.

We provide two theoretical upper bounds on the minimal class-wise sensitivity, which are utilised for accelerating the training of ordinal classifier cascades and allow an exhaustive evaluation of all possible class orders. In this way, ordinal classifier cascades are used as an explorative tool to screen for unknown ordinal dependencies. In our experiments, we give examples for up to 10 different classes resulting in the evaluation of over 3.6 million class orders. Although our algorithm requires pairwise training of the ensemble members, both the bounds and the algorithm are independent of the chosen type of base classifier, the binary training type and might be transferred to alternative ensembles.

Our experiments on the artificial data showed that only if any ordinality is reflected in the feature space possible sets of candidate orders are returned. If there is no ordinal sequence reflected no cascade passes the bound of 0.5. No order was detected for non-ordinal datasets. Always the correct order and its reverse were found as dominant order if cascades were returned for the artificial ordinal datasets.

For all datasets independent of the chosen standard deviation, at least 80% of all candidate cascades could be rejected due to minimal class-wise sensitivities lower than 50%. However, although the procedure can reconstruct the correct class order for all datasets, alternative ordinal class structures might be detected. In our experiments, these alternatives differ from the assumed class order in the position of the last two classes. A reason might be the lower number of constraints for these classes.

For biological applications, we evaluated our method on observable ordinal phenotypes for which a reflection in gene expression levels can be assumed. For three different model organisms we analysed developmental stages characterised by their morphology (*D. melanogaster*)^1^, age (*Danio rerio*)^2^ and number of C-lineage cells (*C. elegans*)^4^. For *C. elegans* also the sampling time points were used in the analysis.

Our screening procedure allowed to reveal ordinal structures within the gene expression profiles of all three model organisms. The hypothesised time relation or its inverse is always included in the set of best-performing cascades. In three out of those four datasets the hypothesised relation dominates with a performance gap towards all other cascades. This strongly indicates a reflection of these orderings in the profiles.

For the *Danio rerio* dataset (*d*_5_) two cascades rank first before a performance gap. There is a swap observed between the two youngest embryo phenotypes. This might be caused either by a data intrinsic reason that those classes are not distinct enough, as staging by days post fertilization has been shown to exhibit high variation in growth rate^28^, or by the technical aspect of the lower number of constraints for later classes.

In contrast to the developmental processes, of which the order of stages is tightly regulated by a genetic program^5,29^, ageing is influenced by multiple factors^30^. Nevertheless we resulted in comparable results for the dataset that measures age related gene expression changes. On the human muscle adaptation dataset 91.7% of all candidate orders were rejected. Among the remaining two candidates the expected order and only one false positive can be found. For non-ordinal phenotypes, as given by the collection of distinct cancer cell lines, no candidate cascades were observed. This indicates that ordinal relations are not a common phenomenon among multiple phenotypes.

Our method can, however, not only be used to confirm proposed hypotheses but also to explore the feature space for potential ordinal structures. This might become relevant if the relation is not easily accessible due to sampling. In surgery, for example, histologically distinguishable tissue regions can be defined in the same biopsy or in single-cell experiments various cell types are extracted from one sample. Within these feature spaces our procedure allows for screening of total ordinal cascades and additionally of ordinal sub-cascades embedded in a larger set of non-ordinal classes. It can hence be used to screen for intrinsic molecular ordinal structures and hypothesis relational axes, which might not be detected in a standard multi-class analysis.

## Methods

We will use following notation throughout the description of the methodology behind the algorithm. An object is represented by a vector of real-valued measurements **x** = (*x*^(1)^, …, *x*^(*n*)^)^*T*^ ∈ ℝ^*n*^. Each object is assumed to be categorisable in one out of $|{\mathscr{Y}}|\ge 2$ predefined classes $y\in {\mathscr{Y}}$, where ${\mathscr{Y}}={\{{y}_{i}\}}_{i=1}^{|{\mathscr{Y}}|}$ denotes the space of all class labels. The general classification task will be to identify a function mapping *c*, a classifier, that allows the accurate prediction of the class labels of new unseen objects $c\,:\,{{\mathbb{R}}}^{n}\to {\mathscr{Y}}=\{{y}_{1},\ldots ,{y}_{|{\mathscr{Y}}|}\}.$

As quality measures, we utilise the conditional prediction rates of *c*. These estimate the probability of classifier *c* to predict the class label *y*_*j*_ for samples of class *y*_*i*_ based on a set of test samples ${{\mathscr{X}}}_{i}$. In its basic version, a conditional prediction rate can be calculated as

$${p}_{c}({y}_{j}|{{\mathscr{X}}}_{i})={\frac{1}{|{{\mathscr{X}}}_{i}|}}_{x\in {{\mathscr{X}}}_{i}}\sum {{\mathbb{I}}}_{[c(x)={y}_{j}]},$$

where ${\mathbb{I}}$_[⋅]_ denotes the indicator function. Other (re-) sampling strategies might be used for determining conditional prediction rates. However, they will not alter the theoretical characteristics discussed in this work. We distinguish between three types of conditional prediction rates: 1. *sensitivities* if *y*_*i*_ = *y*_*j*_ and ${y}_{i},{y}_{j}\in {\mathscr{Y}}$, 2. *confusions* if *y*_*i*_ ≠ *y*_*j*_ and ${y}_{i},{y}_{j}\in {\mathscr{Y}}$, 3. *external rates* if ${y}_{i}\notin {\mathscr{Y}}$ and ${y}_{j}\in {\mathscr{Y}}$.

While (class-wise) sensitivities and confusions build the standard quality measures of a confusion matrix^31^, the external rates describe the classifiers behaviour on foreign classes. They will especially be of interest when dealing with different label spaces.

In the basic multi-class classification scenario, a classifier is typically adapted in a data-driven training procedure based on a set of training samples ${{\mathscr{S}}}_{tr}={\{({{\bf{x}}}_{i},{y}_{i})\}}_{i=1}^{|{{\mathscr{S}}}_{tr}|}$. The basic assumption of this scenario are pairwise distinct classes ${y}_{1}\ne \ldots \ne {y}_{|{\mathscr{Y}}|},$ which can be separated in the chosen feature space. In the ordinal classification scenario it is additionally assumed that the labels in ${\mathscr{Y}}$ are totally ordered ${y}_{(1)}\prec \,\ldots \,\prec {y}_{(|{\mathscr{Y}}|)}.$ In this context, the symbol ${y}_{(i)}$ denotes the $i$th class of the order. We utilise the symbol $\prec$ to indicate that the order relationship is only known for the label space. It is unclear, if this relationship is reflected by the chosen measurements. Nevertheless, ordinal classifiers rely on this assumption. The order of the classes is utilised for guiding the construction of the decision regions. It is provided as additional information to the training algorithm.

### Ordinal classifier cascades

In the following, we will discuss ordinal classifier cascades of type

1 $${h}_{i,j}:{{\mathbb{R}}}^{n}\to \{{y}_{(i)},{y}_{(i+1)},\ldots ,{y}_{(j+1)}\}.$$

The cascade *h*_*i*,*j*_ can be seen as a late-aggregation multi-classifier system^32^, where indices $1\le i\le j < |{\mathscr{Y}}|$ indicate the base classifiers of the corresponding ensemble ${\mathcal E} =\{{c}_{(i)},\ldots ,{c}_{(j)}\}$. The members of the ensemble are designed for separating two neighboured classes *c*_(*i*)_:ℝ^*n*^ → {*y*_(*i*)_,*y*_(*i*+1)_}.

An ordinal cascade will be called a *full cascade* if it is designed for predicting all labels of the label space ${\mathscr{Y}}$. Full cascades will be denoted by $h={h}_{1,|{\mathscr{Y}}|-1}$. Other ordinal cascades will be called *partial cascades*.

The fusion strategy of an ordinal cascade can be interpreted as a sequence of logical conjunctions of its base classifiers

2 $${h}_{i,j}({\bf{x}})=\{\begin{array}{ccc}{y}_{(i)} & {\rm{i}}{\rm{f}} & {c}_{(i)}={y}_{(i)}\\ {y}_{(k)} & {\rm{i}}{\rm{f}} & (\mathop{\wedge }\limits_{i\le l < k\le j}({c}_{(l)}({\bf{x}})={y}_{(l+1)}))\wedge {c}_{(k)}({\bf{x}})={y}_{(k)}\\ {y}_{(j+1)} & {\rm{e}}{\rm{l}}{\rm{s}}{\rm{e}} & \mathop{\mathop{\wedge }\limits_{l=i}}\limits^{j}({c}_{(l)}({\bf{x}})={y}_{(l+1)})\end{array}.$$

**Figure 2:**
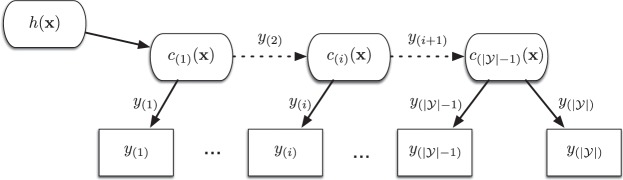
Structure of a full ordinal classifier cascade *h*. The cascade consists of a fixed order of base classifiers *c*_(*i*)_, $1\le i < |{\mathscr{Y}}|$. A sample **x** is passed sequentially from one base classifier to another. If a base classifier *c*_(*i*)_(**x**) predicts class label *y*_(*i*)_, the procedure stops and the ensemble *h* predicts *y*_(*i*)_. Otherwise the ensemble *h* predicts ${y}_{(|{\mathscr{Y}}|)}$.

A scheme of this architecture can be found in Fig. 2. For classifying a sample **x**, the ensemble members *c*_(*k*)_(**x**) are evaluated sequentially according to the assumed order of classes. If a base classifier *c*_(*k*)_(**x**) predicts its first class label *y*_(*k*)_, the procedure stops and *h*_*i*,*j*_(**x**) = *y*_(*k*)_. If it predicts class label *y*_(*k*+1)_ the sample is passed to the subsequent base classifier *c*_(*k*+1)_. This fusion scheme implies following three characteristics on *h*_*i*,*j*_:

1. Each class *y*_(*k*)_, *i* < *k* < *j* + 1 can be predicted by two base classifiers.
2. The lowest class *y*_(*i*)_ can only be predicted by the first classifier *c*_(*i*)_(**x**). The highest classes *y*_(*j*+1)_ can only be predicted by the last classifier *c*_(*j*)_(**x**).
3. A sample **x** will only be passed to a base classifier *c*_(*k*)_(**x**), 1 < *k* ≤ *j*, if all its predecessors *c*_(*l*)_(**x**), *l* < *k*, decide for their second class *y*_(*l*+1)_.

Training algorithms for ordinal classifier cascades mainly focus on the training of the base classifiers. In the following, we utilise a pairwise inductive training, in which the training set ${{\mathscr{S}}}_{(k)}$ of a base classifier *c*_(*k*)_ consists of the samples of classes *y*_(*k*)_ and *y*_(*k*+1)_

3 $${{\mathscr{S}}}_{(k)}=\{({\bf{x}},y)|({\bf{x}},y)\in {{\mathscr{S}}}_{tr},y\in \{{y}_{(k)},{y}_{(k+1)}\}\}.$$

In a previous study, this type of training showed to induce the highest susceptibility to incorrect assumptions on the class order^23^.

### Upper bounds on class-wise sensitivities

The structural properties of the ordinal classifier cascade allow for the construction of upper limits on their empirical class-wise sensitivities. These bounds are based on the training of the cascade’s base classifiers and postulated in Theorem 1. Although this theorem is formulated for full cascades, the corresponding bounds can directly be applied for partial cascades.

**Theorem 1** *Let h denote an ordinal classifier cascade* $h:{{\mathbb{R}}}^{n}\to {\mathscr{Y}}=\{{y}_{(1)},\ldots ,{y}_{(|{\mathscr{Y}}|)}\}$ *with base classifiers* ${\mathcal E} =\{{c}_{(1)},\ldots ,{c}_{(|{\mathscr{Y}}|-1)}\}$. *Let furthermore* ${{\mathscr{X}}}_{(i)}$ *be a non-empty set of samples of class y*_(*i*)_. *Then the sensitivity of h for y*_(*i*)_ *is limited by*

4 $${p}_{h}({y}_{(i)}|{{\mathscr{X}}}_{(i)})\le {p}_{{c}_{(i)}}({y}_{(i)}|{{\mathscr{X}}}_{(i)})$$

5 $${p}_{h}({y}_{(i)}|{{\mathscr{X}}}_{(i)})\le \mathop{{\rm{\min }}}\limits_{k < i}{p}_{{c}_{(k)}}({y}_{(k+1)}|{{\mathscr{X}}}_{(i)}).$$

*Proof*. The theorem is a direct consequence of Lemmata 1 and 2 (see Supplementary).

Theorem 1 states that the sensitivities of an ordinal classifier cascade *h* can be upper bounded by several conditional prediction rates of its base classifiers. For class *y*_(*i*)_, the sensitivity of the cascade is limited by the corresponding sensitivity of its *i*th base classifier *c*_(*i*)_ (Eq. 4). It is also bounded by the predictions of all previous base classifiers *c*_(*k*)_, *k* < *i* (Eq. 5). A sample of class *y*_(*i*)_ will not be classified correctly, if it is classified as *y*_(*k*)_ by *c*_(*k*)_. The sensitivity of the cascade for class *y*_(*i*)_ is therefore also limited by the conditional prediction rate of *c*_(*k*)_ for predicting class label *y*_(*k*+1)_ for samples of class *y*_(*i*)_. A detailed theoretical proof can be found in the Supplementary.

### Detection of ordinal class structures

Ordinal classifier cascades can be used for detecting wrong assumptions about the ordinality of the real class structures. Due to their susceptibility, these classifiers will fail when the real feature structures reflect a different class order or no class order at all. In a screening process, ordinal classifier cascades can be used for revealing unknown ordinal class structures. We have proposed the minimal class-wise sensitivity p^*^ of an ordinal classifier cascade as a measure of the correctness of the assumed class order ${p}^{\ast }={{\rm{\min }}}_{1\le i\le |{\mathscr{Y}}|}{p}_{h}({y}_{(i)}|{{\mathscr{X}}}_{(i)}).$ A sensitivity threshold *t* ≤ p^*^ is used for determining whether an ordinal class structure can be assumed or not. The criterion can be evaluated for each order of the classes in ${\mathscr{Y}}$.

The findings of Theorem 1 allow an alternative evaluation of this criterion. As a direct consequence of Theorem 1, the value of p^*^ can again be upper bounded by conditional prediction rates of the base classifiers

6 $$\forall i:\,{p}^{\ast }\le {p}_{h}({y}_{(i)}|{{\mathscr{X}}}_{(i)})\le {p}_{{c}_{(i)}}({y}_{(i)}|{{\mathscr{X}}}_{(i)})$$

7 $$\forall i:\,{p}^{\ast }\le {p}_{h}({y}_{(i)}|{{\mathscr{X}}}_{(i)})\le \mathop{{\rm{\min }}}\limits_{k < i}\,{p}_{{c}_{(k)}}({y}_{(k+1)}|{{\mathscr{X}}}_{(i)}).$$

Ordinal classifier cascades that are based on wrong assumptions about the ordinality of the classes can therefore be sorted out by the training of the corresponding base classifiers. A graphical illustration describing this sorting out based on a four class example and dependent on Eqs 6 and 7 can be found in Supplementary Fig. S4.

Coupled to a pairwise inductive training of the base classifiers (Eq. 3) the bounds of Theorem 1 can reduce complexity of screens for ordinal structures. As the training of a base classifier *c*_(*i*)_ is only based on the samples of classes *y*_(*i*)_ and *y*_(*i*+1)_, it is no longer dependent on the position of the base classifiers within the cascade *h*. Cascades trained on different orders of ${\mathscr{Y}}$ will therefore consist of common building blocks. The exhaustive training of all $|{\mathscr{Y}}|!$ cascades, each consisting of $|{\mathscr{Y}}|-1$ base classifiers, can therefore be accelerated by precalculating and evaluating all possible $(|{\mathscr{Y}}|-1)|{\mathscr{Y}}|$ base classifiers *c*_*i*,*j*_:ℝ^*n*^ → {*y*_*i*_, *y*_*j*_}. Note that symbols *y*_*i*_, *y*_*j*_ and *c*_*i*,*j*_ no longer rely on an assumed class order.

In any case, the complexity of the exhaustive evaluation is mainly determined by the training and evaluation complexity of the base classifiers. A comparison of the precalculation scheme and a de novo calculation of all cascades in dependency on the numbers of classes $|{\mathscr{Y}}|$ can be found in Table 2. For the presented numbers we assume an evaluation via a single training-test split. For three classes, the de novo strategy already requires twice the number of base classifier trainings and evaluations than the precalculation strategy. For ten classes, the de novo strategy trains more than 3⋅10^7^ base classifiers while the precalculation scheme only demands 90 base classifiers. The number of base classifier trainings and evaluations might be increased by a constant factor if resampling strategies are applied.

**Table 2:** A comparison between the precalculation strategy and a de novo calculation is given.

Number of Classes	Number of Base Classifiers	
	Precalculation	De Novo Training
${\boldsymbol{|}}\pmb{\mathscr{Y}}{\boldsymbol{|}}$	${\boldsymbol{(}}{\boldsymbol{|}}\pmb{\mathscr{Y}}{\boldsymbol{|}}-{\bf{1}}{\boldsymbol{)}}{\boldsymbol{|}}\pmb{\mathscr{Y}}{\boldsymbol{|}}$	${\boldsymbol{(}}{\boldsymbol{|}}\pmb{\mathscr{Y}}{\boldsymbol{|}}-{\bf{1}}{\boldsymbol{)}}{\boldsymbol{|}}\pmb{\mathscr{Y}}{\boldsymbol{|}}{\boldsymbol{!}}$
2	2	2
3	6	12
4	12	72
5	20	480
10	90	>3⋅10^7^
20	380	>4⋅10^19^
30	870	>7⋅10^33^

Number of base classifiers required for an exhaustive training of all $|{\mathscr{Y}}|!$ ordinal classifier cascades is shown. The table gives the number of base classifiers that are required to be trained and evaluated within the screening process based on a single training-test training.

The following quality measures are needed for the application of Theorem 1:

8 $$F{C}_{i,j}={p}_{{c}_{i,j}}({y}_{i}|{{\mathscr{X}}}_{i})\,{\rm{and}}\,S{C}_{i,j}(r)={p}_{{c}_{i,j}}({y}_{j}|{{\mathscr{X}}}_{r}).$$

Here, *FC*_*i*,*j*_ denotes the class-wise sensitivity of *c*_*i*,*j*_ for predicting its first class label *y*_*i*_. The term *SC*_*i*,*j*_(*r*) denotes the conditional prediction rates of *c*_*i*,*j*_ for samples of class *y*_*r*_ that are classified as *y*_*j*_. Both quantities can be precalculated and memorised for all binary base classifiers (Table 3).

**Table 3:** Training table of binary classifiers *c*_*i*,*j*_ in a multi-class scenario ($|{\mathscr{Y}}| > 2$).

*c*_*i*,*j*_(x)		First Class *FC*_*i*,*j*_	Second Class *SC*_*i*,*j*_(*r*)				
class *i*	class *j*	*y*_*i*_	*y*_1_	…	*y*_*r*_	…	${y}_{|{\mathscr{Y}}|}$
$\vdots$	$\vdots$	$\vdots$	$\vdots$		$\vdots$		$\vdots$
*y*_*i*_	*y*_*j*_	${p}_{{c}_{i,j}}({y}_{i}\,|\,{{\mathscr{X}}}_{i})$	$\vdots$	${p}_{{c}_{i,j}}({y}_{j}\,|\,{{\mathscr{X}}}_{r})$			$\vdots$
$\vdots$	$\vdots$	$\vdots$	$\vdots$		$\vdots$		$\vdots$

Each classifier *c*_*i*,*j*_ is trained on the samples of classes ${y}_{i},{y}_{j}\in {\mathscr{Y}}$ and evaluated on the samples of all classes in ${\mathscr{Y}}$. The sensitivity of *c*_*i*,*j*_ on detecting samples of *y*_*i*_ is denoted by *FC*_*i*,*j*_. The conditional prediction rates of *c*_*i*,*j*_ of predicting class *y*_*j*_ for samples of class *y*_*r*_ is given by *SC*_*i*,*j*_(*r*).

### The CASCADES algorithm

**Figure 3:**
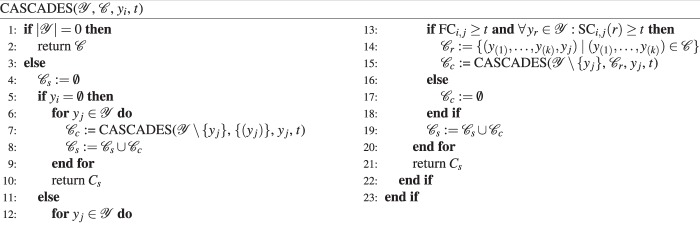
CASCADES algorithm for an exhaustive screening of all orders of class labels $y\in {\mathscr{Y}}$. The function parameters are the set of class labels ${\mathscr{Y}}$, a set of candidate cascades ${\mathscr{C}}$, the threshold *t* and *y*_*i*_ which specifies the class label that was selected in the previous recursion.

We propose the recursive enumeration scheme ${\rm{CASCADES}}({\mathscr{Y}},{\mathscr{C}},{y}_{i},t)$ for the exhaustive training of all orders of ${\mathscr{Y}}$ (Fig. 3). It can be seen as a filter routine that rejects ordinal cascades that will not achieve a minimal class-wise sensitivity *t* ≤ p^*^ according to the bounds of Theorem 1. The remaining cascades will be returned as a set of candidates ${\mathscr{C}}$.

The CASCADES algorithm is based on the evaluation of an extended confusion table as shown in Table 3. It will replace the training procedure of each base classifier (training-test split or resampling strategy) by looking up $|{\mathscr{Y}}|-k$ conditional prediction rates, where *k* is the base classifiers position within the cascade. As there exist $$(\begin{array}{l}|{\mathscr{Y}}|\\ k+1\end{array})$$ possibilities of constructing subcascades (prefixes) of *k* base classifiers, at most $$f(|{\mathscr{Y}}|):=\mathop{\sum }\limits_{k=2}^{|{\mathscr{Y}}|}\,k(\begin{array}{l}|{\mathscr{Y}}|\\ k+1\end{array})$$ comparisons are required in a worst case scenario. Nevertheless, this number will rapidly break down by utilising early stopping criteria.

A single cascade is represented as an ordered vector of class labels $({y}_{(1)},\ldots ,{y}_{(|{\mathscr{Y}}|)})\in {\mathscr{C}}.$ Each candidate cascade is constructed sequentially. It is extended by a new class label in each recursive call of the algorithm. The construction stops, if the performance measures of the current base classifier falls under the chosen threshold *t*. In this case the candidate cascade is rejected.

The sequential extension of a partial cascade *h*_1,*k*−1_ improves the runtime of the exhaustive search. If the *k*th (candidate) base classifier does not fulfil the minimal criteria, all full ordinal cascades that utilise *h*_1,*k*_ as prefix can be withdrawn. This corresponds to $(|{\mathscr{Y}}|-k-1)!$ full ordinal cascades. A single early stopping will reduce the number of lookups by at least $f(|{\mathscr{Y}}|-k)$.

The algorithm is initialised with the full set of labels ${\mathscr{Y}}$, an empty set of candidate cascades ${\mathscr{C}}=\varnothing$, the chosen threshold *t* and *y*_*i*_ = ∅. The parameter *y*_*i*_ will later on indicate the class label selected in the previous recursion. In each recursion, the class labels ${y}_{j}\in {\mathscr{Y}}$ are tested as possible extensions of the candidate cascades in ${\mathscr{C}}$. If FC_*i*,*j*_ ≥ *t* and $\forall \,{y}_{r}\in {\mathscr{Y}}:{{\rm{SC}}}_{i,j}(r)\ge t$ the current base classifier *c*_*i*,*j*_ fulfils the bounds on p^*^. In this case, class label *y*_*j*_ can be added to the current candidate cascade and can be removed from the set of remaining labels ${\mathscr{Y}}$. The next base classifier will be chosen by a recursive call CASCADES(${\mathscr{Y}}\backslash \{{y}_{j}\}$, ${{\mathscr{C}}}_{r}$, *y*_*j*_, *t*). If the current base classifier does not fulfil the minimal criteria, the corresponding (partial) candidate cascades are erased and an empty set ∅ is returned. All suitable candidate cascades are collected at the end of the recursive call. Although the algorithm CASCADES rejects cascades with too low minimal class-wise sensitivities, the remaining candidates are not guaranteed to fulfil the minimal criterion *t* ≤ p^*^. Each of the final candidates must therefore be cross-checked by an evaluation of the full cascade. CASCADES can directly be applied for the evaluation of partial ordinal cascades. By replacing the initial set of class labels ${\mathscr{Y}}$ by a subset ${\mathscr{Y}}\text{'}\subset {\mathscr{Y}}$, the algorithm will evaluate all orders of the class labels in ${\mathscr{Y}}\text{'}$.

### Datasets

An overview of the characteristics of all datasets can be found in Supplementary Table S1. The datasets *d*_5_–*d*_9_ were collected from the gene expression omnibus repository^33^ (GSE13371, GSE47881, GSE2180, GSE32474) and processed using the robust multi-array average (rma) normalisation as implemented in the affy package^34^. For *d*_4_ the processed data was downloaded.

#### Linear dataset (*d*_1_)

For a first series of experiments, the centroid of the *i*th class *y*_*i*_ is chosen as ${{\bf{m}}}_{{y}_{i}}={(i,i)}^{T}$. In this way, the class centroids lie on a line.

#### Curved dataset (***d***_2_)

For the second dataset, the class centroids were chosen depending on their predecessors. ${{\bf{m}}}_{{y}_{i}}={({m}_{{y}_{i}}^{(1)},{m}_{{y}_{i}}^{(2)})}^{T}={({m}_{{y}_{i-1}}^{(1)}+{u}_{i}^{(1)},{m}_{{y}_{i-1}}^{(2)}+{u}_{i}^{(2)})}^{T}$, where ${u}_{i}^{(1)},{u}_{i}^{(2)} \sim {\mathscr{U}}(0.5,2)$. As a starting point ${{\bf{m}}}_{{y}_{0}}={(0,0)}^{T}$ was chosen. This dataset has a curved shape.

#### Non-ordinal dataset (***d***_3_)

The third artificial dataset is designed to be non-ordinal. The centers of the classes are arranged on a predefined two dimensional grid in the range [1, 4]^2^ (Supplementary Table S2).

#### Drosophila melanogaster (***d***_4_)

The drosophila dataset generated by Arbeitman *et al*.^1^ consists of gene expression profiles of the fruit fly *Drosophila melanogaster*. These profiles consist of 4028 measurements and were collected at different points in time during the life cycle of the model organism. They can be categorised according to the developmental stages of *Drosophila melanogaster*: $embryo\prec larva\prec pupa\prec adult.$ Overall the dataset contains profiles for 31 *embryos*, 10 *larvae*, 18 *pupae* and 8 *adults*. We use the natural order of the developmental stages as ordinal class labels for our experiment.

#### Danio rerio (***d***_5_)

The dataset collected by Toyama *et al*.^2^ consists of gene expression profiles of the pineal glands of zebrafish (*Danio rerio*). The expression profiles were collected at five different time points: $$embry{o}_{1}\prec embry{o}_{2}\prec embry{o}_{3}$$$$\prec adul{t}_{1}\prec adul{t}_{2},$$ where *embryo*_1_-*embryo*_3_ were collected 3, 5 and 10 days after birth and *adult*_1_ and *adult*_2_ were collected at an age of 3 months and 1–2 years. The dataset comprises 12–15 samples for each class. The age of the samples will be used as class order.

#### Human muscle adaptation (***d***_6_)

Philips *et al*.^3^ have compared the transcriptome of human muscle cells before and after 20 weeks of supervised resistance-exercise training (RET). The corresponding dataset consists of paired gene expression profiles. For our experiments, the data was categorised into four classes according to the age (in years) of the participants: $$ag{e}_{1}\prec ag{e}_{2}\prec ag{e}_{3}\prec ag{e}_{4}.$$ The class labels denote age intervals of $$[20;40)$$ years (18 samples), $$[40;60)$$ years (38 samples), $$[60;70)$$ years (16 samples) and $$[70;80)$$ years (16 samples). In order to avoid overoptimistic results, we ensured that the profiles of a subject are not used for training and testing the classifier simultaneously.

#### Caenorhabditis elegans (***d***_7_ and ***d***_8_)

Baugh *et al*.^4^ analysed the influence of the homeodomain protein PAL-1 of the C-lineage-specific gene regulatory network in the model organism *C. elegans*. They gathered gene expression data of samples of wild-type embryos and mutant embryos with additional C blastomeres, as well as on samples of mutants without any C blastomeres. For our experiments we used data of the C-cell-free organisms, taken at 10 points in time after the 4-cell-stage of the embryo. We labelled these samples in two different ways. In the first experiment (*d*_7_) the samples were labelled according to the developmental stages proposed in the original publication: $stag{e}_{1}\prec stag{e}_{2}\prec stag{e}_{3}\prec stag{e}_{4}\prec stag{e}_{5},$ where 0 and 23 minutes samples were merged in the *stage*_1_ class, 41 and 53 minutes samples in *stage*_2_, and samples taken at 66, 83 and 101 minutes after the 4-cell stage in the class *stage*_3_. *stage*_4_ only consists of samples taken at 122 minutes, and *stage*_5_ contains time points 143 and 186 minutes. In the second experiment (*d*_8_), the points in time were analysed solely: ${t}_{1}\,\prec \,{t}_{2}\,\prec \,{t}_{3}\,\prec {t}_{4}\,\prec \,{t}_{5}\,\prec \,{t}_{6}\,\prec {t}_{7}\,\prec \,{t}_{8}\,\prec \,{t}_{9}\,\prec \,{t}_{10}.$

#### Various cancer cell lines (***d***_9_)

Pfister *et al*.^27^ collected gene expression profiles from cell lines that derived from 9 different cancer tissue types (*breast* (15 samples), *central nervous system* (18 samples), *colon* (21 samples), *leukemia* (18 samples), *melanoma* (26 samples), *non-small cell lung* (26 samples), *ovarian* (21 samples), *prostate* (6 samples), *renal (23 samples)*). In contrast to *d*_1_–*d*_8_, the classes of this dataset are not assumed to fulfil an ordinal relationship as each group originates from a different tissue type: *line*_1_ ≠ *line*_2_ ≠ *line*_3_ ≠ *line*_4_ ≠ *line*_5_ ≠ *line*_6_ ≠ *line*_7_ ≠ *line*_8_ ≠ *line*_9_.

## Supplementary information

Supplementary Info

## Data Availability

The drosophila dataset is available from http://flygenome.yale.edu/Lifecycle/. The other datasets are available from the GEO repository https://www.ncbi.nlm.nih.gov/gds: GSE13371, GSE47881, GSE2180, GSE32474.
