# Supplementary material for: Assessing phenotype order in molecular data
Source: Sci Rep. 2019 Aug 13;9:11746. doi: 10.1038/s41598-019-48150-z (PMC6692304; doi:10.1038/s41598-019-48150-z)
Supplement: Supplementary file 1 — Supplementary Info [file 41598_2019_48150_MOESM1_ESM.pdf]

Supplementary Information
Assessing phenotype order in molecular data

Ludwig Lausser[1,+], Lisa M. Schäfer[1,+], Lyn-Rouven Schirra[1,+], Robin Szekely[1], Florian Schmid[1], and Hans A. Kestler[1,*]

[1]Institute of Medical Systems Biology, Ulm University, 89069 Ulm, Germany
[*]hans.kestler@uni-ulm.de
[+]these authors contributed equally to this work

Proof of Theorem 1

The following provides the proof of Theorem 1. It is based on Lemmata S1 and S2 which are derived from the more general Theorems S1 and S2.

Theorem S1. *Let h denote a full ordinal classifier cascade*

$$h : \mathbb{R}^n \longrightarrow \mathscr{Y} = \left\{y_{(1)}, \ldots, y_{(|\mathscr{Y}|)}\right\}. \tag{S1}$$

Let furthermore i, j,k denote indices with $1 \leq i \leq j \leq |\mathscr{Y}| - 1$ *and* $j < k \leq |\mathscr{Y}|$*. For each partial ordinal classifier cascade* $h_{i,j}$ *and for each set of samples* $\mathscr{X}$

$$p_h(y_{(k)} \,|\, \mathscr{X}) \leq p_{h_{i,j}}(y_{(j+1)} \,|\, \mathscr{X}). \tag{S2}$$

Proof. Theorem S1 states that a partial cascade $h_{i,j}$ predicts its highest class label $y_{(j+1)}$ more often than the full cascade h predicts any subsequent class label $y_{(k)}$, $k \geq j+1$. Let in the following M denote the number of evaluations of the chosen type of resampling experiment on $\mathscr{X}$. The number of samples (more precisely instances) of $\mathscr{X}$ classified as $y_{(l)}$ by the full cascade is then given by

$$m_{(l)} = M \cdot p_h(y_{(l)} \,|\, \mathscr{X}). \tag{S3}$$

The corresponding number for the partial ordinal cascade will be denoted as

$$\dot{m}_{(l)} = M \cdot p_{h_{i,j}}(y_{(l)} \,|\, \mathscr{X}). \tag{S4}$$

Case $i = 1$: If both cascades start with the classifier $c_{(1)}$, the full cascade can be seen as an extension of the partial cascade. The same samples will be classified as $y_{(1)}, \ldots, y_{(j)}$. For these classes, $m_{(1)} = \dot{m}_{(1)}$, ..., $m_{(j)} = \dot{m}_{(j)}$. The remaining samples will be classified as $y_{(j+1)}$ by the partial cascade while the full cascade is able to categorize the remaining samples as $y_{(j+1)}, \ldots, y_{(|\mathscr{Y}|)}$

$$\dot{m}_{(j+1)} = M - \sum_{l=1}^{j} m_{(l)} = \sum_{l=j+1}^{|\mathscr{Y}|} m_{(l)}. \tag{S5}$$

As all $m_{(i)} \geq 0, \dot{m}_{(j+1)} = m_{(k)}$, holds for at most one k if the full cascade constantly predicts all $y_{(k)}$ for all remaining samples, which is trivially fulfilled for $j+1 = |\mathscr{Y}|$. For a non constant classification of the samples $\dot{m}_{(j+1)} > m_{(k)}$:

Case $i > 1$: In this case we cannot assume that each sample that is classified as $y_{(i)}, \ldots, y_{(j+1)}$ by the partial cascade receives the same class label by the full cascade. However, if a sample passes classes $y_{(1)}, \ldots, y_{(i-1)}$ in the full cascade they will receive class label $y_{(l)}$ if and only if the partial cascade predicts $y_{(l)}$. None of the base classifiers $c_{(1)}, \ldots, c_{(i-1)}$ will predict a class

label $y_{(k)}$, $k \geq j+1$. That is, we can give a lower bound on the number of samples that will receive a class label $y_{(1)}, \ldots, y_{(j)}$ from the full cascade by the number of samples that receive a class label $y_{(i)}, \ldots, y_{(j)}$ from the partial cascade

$$\sum_{l=i}^{j} \dot{m}_{(l)} \leq \underbrace{\sum_{l=1}^{i-1} m_{(l)}}_{\geq 0} + \sum_{l=i}^{j} m_{(l)} \quad \Longleftrightarrow \tag{S6}$$

$$\Longleftrightarrow \quad M - \sum_{l=i}^{j} \dot{m}_{(l)} \geq M - \left(\sum_{l=1}^{i-1} m_{(l)} + \sum_{l=i}^{j} m_{(l)} \right) \tag{S7}$$

$$\Longleftrightarrow \quad \dot{m}_{(j+1)} \geq \sum_{l=j+1}^{|\mathscr{Y}|} m_{(l)} \,. \tag{S8}$$

Similar as before, $m_{(i)} \geq 0, \dot{m}_{(j+1)} = m_{(k)}$ holds for at most one k, if the classifier omits the prediction of class labels

$$y \in \left\{ y_{(1)}, \ldots, y_{(i-1)}, y_{(j+1)}, \ldots, y_{(|\mathscr{Y}|)} \right\} \setminus \left\{ y_{(k)} \right\} \,. \tag{S9}$$

□

Lemma S1. *Let h denote a full ordinal classifier cascade*

$$h : \mathbb{R}^n \longrightarrow \mathscr{Y} = \left\{ y_{(1)}, \ldots, y_{(|\mathscr{Y}|)} \right\} . \tag{S10}$$

with base classifiers $\mathscr{E} = \left\{ c_{(1)}, \ldots, c_{(|\mathscr{Y}|-1)} \right\}$. For each $c_{(i)} \in \mathscr{E}$ and for each k with $i < k \leq |\mathscr{Y}|$

$$p_h(y_{(k)} \,|\, \mathscr{X}) \leq p_{c_{(i)}}(y_{(i+1)} \,|\, \mathscr{X}). \tag{S11}$$

Proof. Lemma S1 is a direct consequence of Theorem S1 as $c_{(i)} = h_{i,i}$. □

Theorem S2. *Let h denote a full ordinal classifier cascade*

$$h : \mathbb{R}^n \longrightarrow \mathscr{Y} = \left\{ y_{(1)}, \ldots, y_{(|\mathscr{Y}|)} \right\} . \tag{S12}$$

Let furthermore i, j, k denote indices with $1 \leq i \leq j \leq |\mathscr{Y}| - 1$ and $i \leq k \leq j$. For each partial ordinal classifier cascade $h_{i,j}$ and for each set of samples $\mathscr{X}$

$$p_h(y_{(k)} \,|\, \mathscr{X}) \leq p_{h_{i,j}}(y_{(k)} \,|\, \mathscr{X}). \tag{S13}$$

Proof. Theorem S2 states that each class label $y_{(k)}$, $i \leq k \leq j$ which is covered by a partial cascade $h_{i,j}$ is predicted at least in the same number of cases by the partial cascade as it is by the full cascade. Let $m_{(l)}$ and $\dot{m}_{(l)}$ be defined as in Equations S3 and S4. As before, we can assume that both the full cascade and the partial cascade will classify the same samples as $y_{(i)}, \ldots, y_{(j)}$ if the full cascade did not predict class labels $y_{(1)}, \ldots, y_{(i-1)}$. The set of remaining samples of the partial cascade is therefore a superset of remaining samples of the full cascade

$$M - \sum_{l=i}^{k-1} \dot{m}_{(l)} \geq M - \left(\sum_{l=1}^{i-1} m_{(l)} + \sum_{l=i}^{k-1} m_{(l)} \right) . \tag{S14}$$

The additional remaining samples of the partial ordinal cascade can increase the number of predictions of class $y_{(k)}$. □

Lemma S2. *Let h denote an full ordinal classifier cascade*

$$h : \mathbb{R}^n \longrightarrow \mathscr{Y} = \left\{ y_{(1)}, \ldots, y_{(|\mathscr{Y}|)} \right\} . \tag{S15}$$

with base classifiers $\mathscr{E} = \left\{ c_{(1)}, \ldots, c_{(|\mathscr{Y}|-1)} \right\}$. For each $c_{(i)} \in \mathscr{E}$ and for each $k \in \{i, i+1\}$

$$p_h(y_{(k)} \,|\, \mathscr{X}) \leq p_{c_{(i)}}(y_{(k)} \,|\, \mathscr{X}). \tag{S16}$$

Proof. Lemma S2 is a direct consequence of Theorem S2 as As $c_{(i)} = h_{i,i}$. □

Dataset Information

Table S1. Overview on utilised datasets. The table shows the datasets analysed in the 10×10 cross-validation experiments. For each dataset, the name, the type of classification problem, the number of classes $|\mathscr{Y}|$, the number of class orders $|\mathscr{Y}|!$, the overall number of samples m, the class-wise number of samples m_i and number of features n is presented. Datasets d_1 to d_3 are artificially generated, d_4 to d_9 are gene expression datasets.

id	name	ordinal	$\lvert\mathscr{Y}\rvert$	$\lvert\mathscr{Y}\rvert!$	m	m_i	n
$\mathbf{d_1}$:	**linear**<br>$y_1 \prec \ldots \prec y_{10}$	yes	10	$\sim 3.6 \cdot 10^6$	1000	$\forall i$: $m_i = 100$	2
$\mathbf{d_2}$:	**curved**<br>$y_1 \prec \ldots \prec y_{10}$	yes	10	$\sim 3.6 \cdot 10^6$	1000	$\forall i$: $m_i = 100$	2
$\mathbf{d_3}$:	**non-ordinal**<br>-	no	10	$\sim 3.6 \cdot 10^6$	1000	$\forall i$: $m_i = 100$	2
$\mathbf{d_4}$:	**drosophila**[1]<br>$embryo \prec larva \prec pupa \prec adult$	yes	4	24	67	$m_1 = 31$, $m_2 = 10$,<br>$m_3 = 18$, $m_4 = 8$	4028
$\mathbf{d_5}$:	**danio rerio**[2]<br>$embryo_1 \prec embryo_2 \prec embryo_3$<br>$\prec adult_1 \prec adult_2$	yes	5	120	69	$m_1 = 14$, $m_2 = 14$, $m_3 = 15$,<br>$m_4 = 12$, $m_5 = 14$	15502
$\mathbf{d_6}$:	**human muscle**[3]<br>$age_1 \prec age_2 \prec age_3 \prec age_4$	yes	4	24	89	$m_1 = 19$, $m_2 = 38$,<br>$m_3 = 16$, $m_4 = 16$	54613
$\mathbf{d_7}$:	**c. elegans (stages)**[4]<br>$stage_1 \prec stage_2 \prec stage_3$<br>$\prec stage_4 \prec stage_5$	yes	5	120	62	$m_1 = 12$, $m_2 = 12$, $m_3 = 19$,<br>$m_4 = 7$, $m_5 = 12$<br>$m_4 = 7$, $m_5 = 12$	22548
$\mathbf{d_8}$:	**c. elegans (time points)**[4]<br>$t_1 \prec t_2 \prec t_3 \prec t_4 \prec t_5 \prec t_6$<br>$\prec t_7 \prec t_8 \prec t_9 \prec t_{10}$	yes	10	$\sim 3.6 \cdot 10^6$	62	$m_1 = 6$, $m_2 = 6$, $m_3 = 6$,<br>$m_4 = 6$, $m_5 = 6$, $m_6 = 8$,<br>$m_7 = 5$, $m_8 = 7$, $m_9 = 5$<br>$m_{10} = 7$	22548
$\mathbf{d_9}$:	**cancer cell lines**[5]<br>-	no	9	362880	174	$m_1 = 15$, $m_2 = 18$, $m_3 = 21$,<br>$m_4 = 18$, $m_5 = 26$, $m_6 = 26$,<br>$m_7 = 21$, $m_8 = 6$, $m_9 = 23$	54613

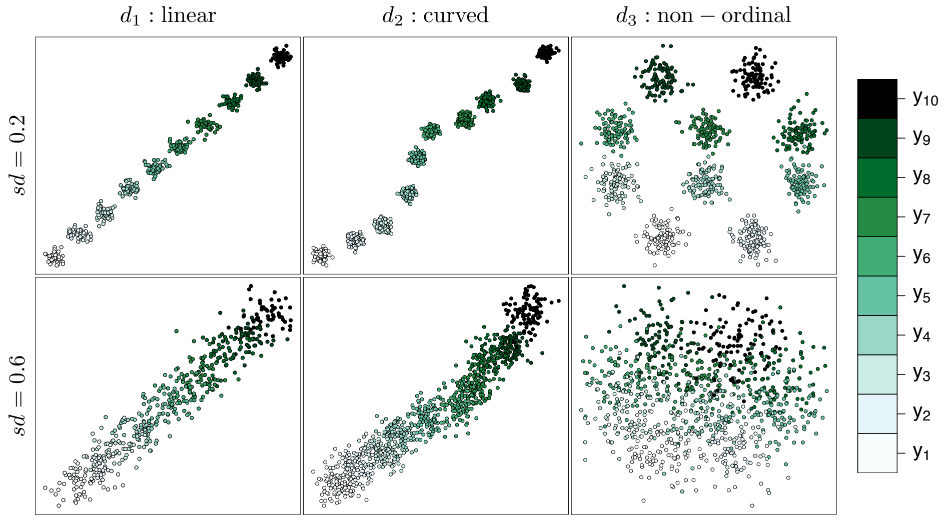

Figure S1. Concept of the artificial datasets. The figure shows examples for the artificially generated datasets d_1 (linear), d_2 (curved) and d_3 (non-ordinal). All datasets are based on $|\mathscr{Y}| = 10$ classes and $m = 100$ samples per class. The upper column gives examples for $sd = 0.2$. The lower one shows examples for $sd = 0.6$.

Table S2. Class centers of artificial dataset d_3 (non-ordinal dataset).

	y_1	y_2	y_3	y_4	y_5	y_6	y_7	y_8	y_9	y_{10}
$\mathbf{m}_y$	$\binom{2}{1}$	$\binom{4}{1}$	$\binom{1}{2}$	$\binom{3}{2}$	$\binom{5}{2}$	$\binom{1}{3}$	$\binom{3}{3}$	$\binom{5}{3}$	$\binom{2}{4}$	$\binom{4}{4}$

Performance dependency on the standard deviation of the artificial results

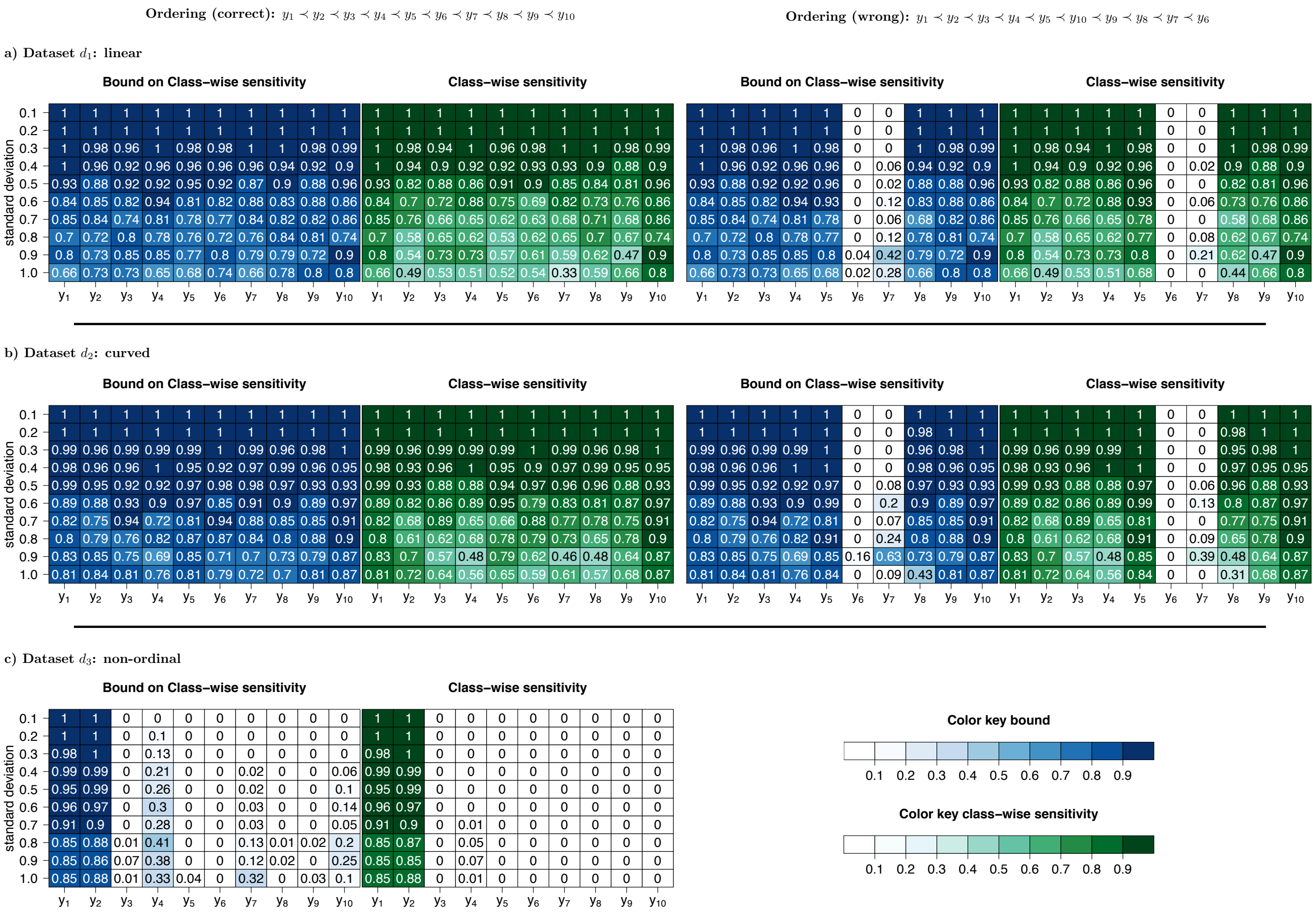

Figure S2. Sensitivities (10×10 CV) gained for the artificial datasets $d_1 - d_3$. The computational upper bounds (blue) and the real class-wise sensitivities (green) are shown. Datasets were generated for standard deviations $sd \in \{0.1, 0.2, \ldots 1.0\}$. For the ordinal datasets d_1 : *linear* and d_2 : *linear* a cascade was trained for the correct class order (left column) and a wrong class order (right column). For the wrong class order, classes y_6 and y_7 are only rarely detected (sensitivities near zero). Similar observations can be made for the non-ordinal dataset d_3.

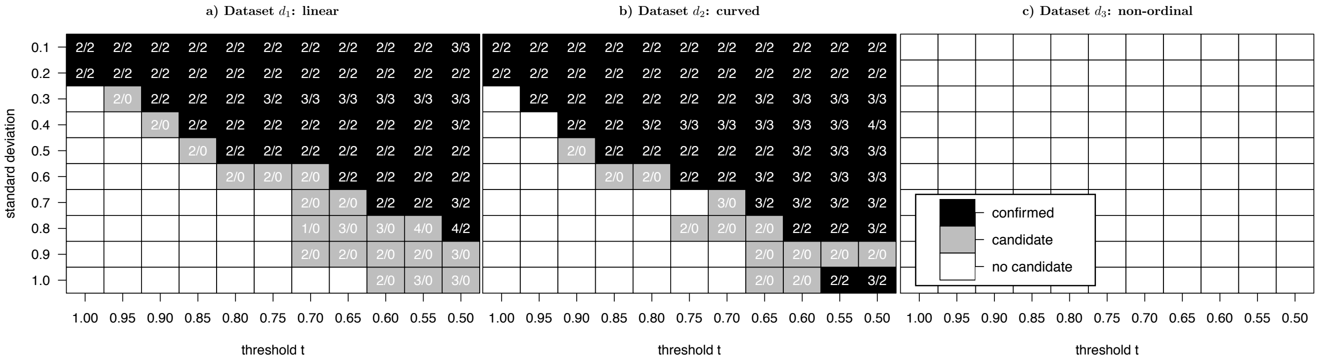

Figure S3. Evaluation of the CASCADES algorithm on the artificial datasets d_1 (linear), d_2 (curved) and d_3 (non-ordinal). Screening experiments were performed for datasets with standard deviations $sd \in \{0.1, 0.2, \ldots, 1.0\}$ and sensitivity thresholds $t \in \{1.00, 0.95, \ldots, 0.50\}$. Each screening experiment comprises all $10! \approx 3.6 \cdot 10^6$ class orderings. For each experiment, the number of *candidate*/*confirmed* cascades is reported, where *candidates* are proposed by the CASCADES algorithm (applied bound 0.5) and *confirmed* cascades achieve a minimal class-wise sensitivity higher than t.

Algorithm

a)

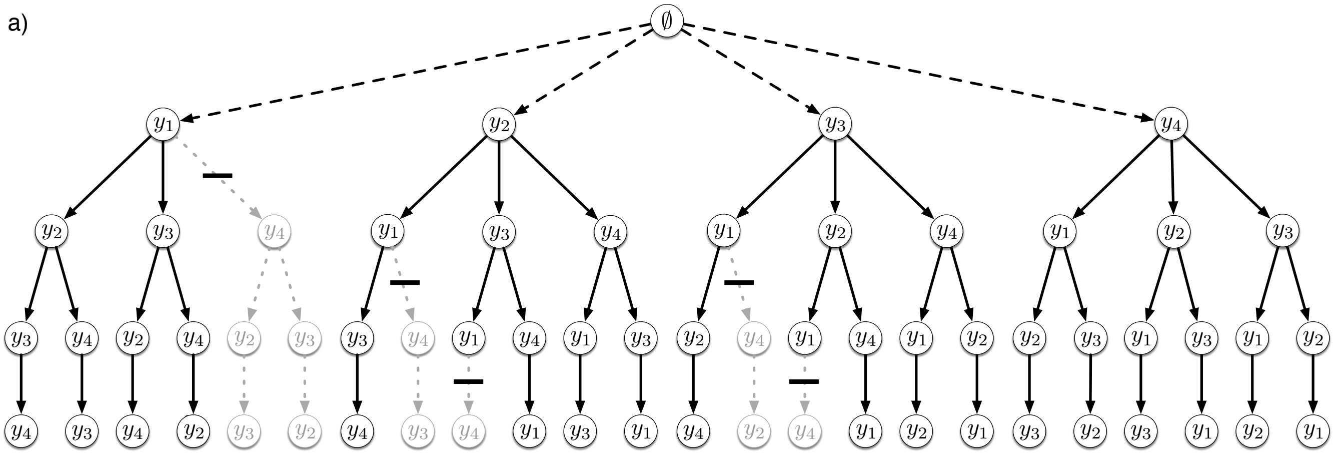

b)

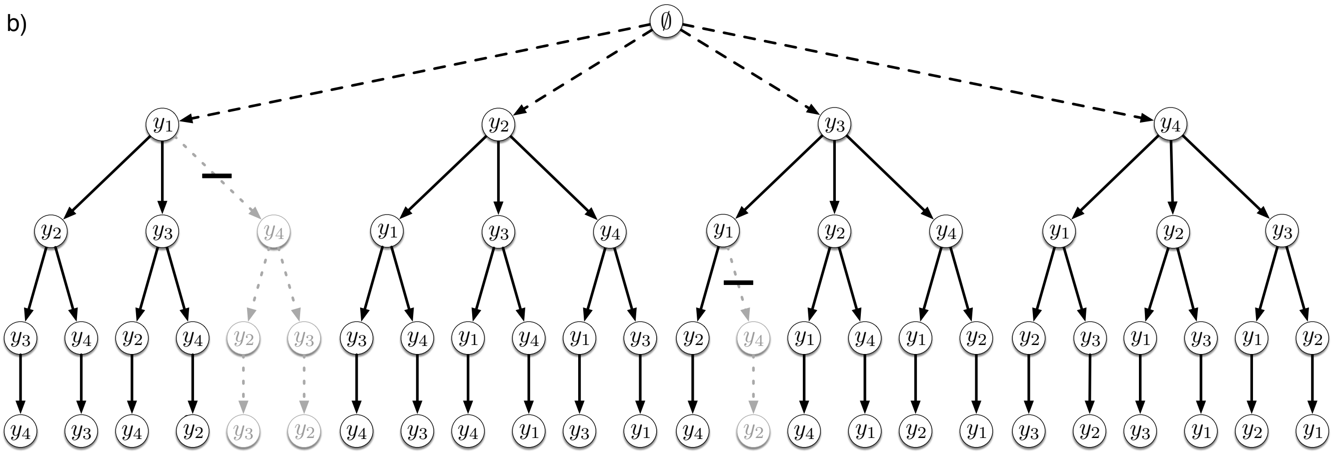

Figure S4. Iterative enumeration of class orderings. An example for four classes (y_1, y_2, y_3, y_4) is shown. A partial class ordering can be extended by adding one of the remaining labels. The base classifiers of the corresponding ordinal classifier cascade are indicated by the solid arrows. If a base classifier does not fulfil the criteria for the minimal class-wise sensitivity (Theorem 1), the subsequent tree of evaluations can be pruned. Panel a gives an example for a too low sensitivity of classifier $c_{1,4}$. Panel b gives an example for a too low conditional prediction rate of $c_{1,4}$ for class y_2.

References

1. Arbeitman, M. N. *et al.* Gene expression during the life cycle of Drosophila melanogaster. *Science* **297**, 2270–2275 (2002).

2. Toyama, R. *et al.* Transcriptome analysis of the zebrafish pineal gland. *Dev. Dyn.* **238**, 1813–1826 (2009).

3. Phillips, B. E. *et al.* Molecular networks of human muscle adaptation to exercise and age. *PLOS Genet.* **9**, 1–15 (2013).

4. Baugh, L. R. *et al.* The homeodomain protein PAL-1 specifies a lineage-specific regulatory network in the C. elegans embryo. *Development* **132**, 1843–1854 (2005).

5. Pfister, T. D. *et al.* Topoisomerase I levels in the NCI-60 cancer cell line panel determined by validated ELISA and microarray analysis and correlation with indenoisoquinoline sensitivity. *Mol. Cancer Ther.* **8**, 1878–1884 (2009).
